# Comment on Hietanen et al. Cytolytic Properties and Genome Analysis of Rigvir^®^ Oncolytic Virotherapy Virus and Other Echovirus 7 Isolates. *Viruses* 2022, *14*, 525

**DOI:** 10.3390/v14092076

**Published:** 2022-09-19

**Authors:** Pēteris Alberts

**Affiliations:** Rigvir, Atlasa iela 7C, LV-1026 Riga, Latvia; peteris.alberts@rigvir.com

## 1. Abstract

In a recent article published in *Viruses* by Hietanen et al. [1], the authors performed a phylogenetic analysis of the full-length nucleotide sequences of six different ECHO-7 virus isolates, some of which had been previously published by the same authors [2]. In addition, the authors tested for cytopathic effect on cells in culture, which partially repeated and extended previous results [3].

Regardless, readers should be advised about the following problems with the article [1], in my opinion.

## 2. About Rebutting of Allegedly Made Claims Regarding the Oncolytic Virus Rigvir and Conclusions and Opinions That Are Not Based on Results

In the Abstract, the authors wrote that “we conclude that Rigvir^®^’s claim of being an effective treatment against multiple different cancers is not warranted under the evidence presented here.” The conclusion does not follow from the results presented, since the article does not provide any clinical results. Preclinical results are obtained to predict effects in the clinical setting. The results in, for example, Table 2 [1] show that all six ECHO-7 strains tested, including Rigvir, showed cytopathic effect in cell lines. In addition, Rigvir has previously been shown to be effective and approved for melanoma [4,5].

In the Introduction, the authors wrote, “Rigvir^®^ claims to” be “free from adverse effects to the patient.” In fact, Rigvir has been shown to be well tolerated and to cause no, if any, serious adverse effects [4].

In the Results, the authors wrote, “These results indicated that Rigvir^®^’s claimed properties could be due to subtle differences in receptor binding as a consequence of unique mutations located on the viral capsid surface”, and in the Discussion, the authors wrote, “individual mutations are difficult to claim as being responsible for such a drastic change in virus behavior, as is claimed for Rigvir^®^.” These are confusing and misleading statements, since it is not clear where the claims would have been made.

In the Discussion, the authors wrote, “it is difficult to conceive the need for consecutive injection during the treatment, since it is likely that the adaptive immune response generated after the first dose will halt the secondary infections”, and “the data regarding sequence variation and receptor use are inconclusive and insufficient for explaining the clinical benefit of Rigvir^®^ and calls for further studies to warrant the use of Rigvir^®^ or other E7 isolates in oncolytic virotherapy”. Usually, the discussion is about the authors’ own results. In addition, Rigvir has previously been shown to be effective in the clinical setting [4,5], and therefore, speculation as to that is overdue.

The authors wrote, “Rigvir^®^ was administered by muscular injections, that is, the effect is claimed to occur systemically”. It is not known where that claim would have been made. Interestingly, intramuscular administration is actually considered to be systemic since it bypasses first-pass metabolism [6].

## 3. About the Use of Outdated, Wrong and Irrelevant Information

In the Discussion, the authors wrote, “the lack of comparative studies against native cell lines, as well as the inclusion of additional clinical E7 isolates, is a major flaw in the analysis of a claimed oncolytic and oncotropic virotherapy drug such as Rigvir^®^”. This statement ignores the fact that studies on normal cells were published in 2018 [3] and that during Rigvir development, approximately 60 different types of viruses were screened, including ECHO 1-19, Coxsackie A1-A19, B1-B5 and a few pathogenic polioviruses [4].

In the Introduction, the authors wrote, “Rigvir^®^’s marketing website has no information about the withdrawal, and still lists the drug being approved in Uzbekistan, Georgia, and Armenia.” This statement is wrong about the Rigvir marketing website [7], since the latter displays correct and updated information at all times.

In the Introduction, the authors wrote, “there have not been conventional clinical trials using Rigvir^®^ https://www.clinicaltrials.gov/, (accessed on 19 February 2022)”. The Rigvir pre-registration clinical studies were performed during the period 1968–1991 [4], while the cited website was launched a decade later in 2000, making the reference to it irrelevant. 

In several instances, the authors wrote that Rigvir would be available at Hope4Cancer Institute https://hope4cancer.com/ (accessed on 19 February 2022). The mentioned cancer institute does not use Rigvir and has not done so for several years, and there is no mention of Rigvir on the cited homepage (accessed 8 March 2022). 

In several instances, the authors wrote that Rigvir has been cell-adapted. Actually, Rigvir has been melanoma-adapted, since during development Rigvir was selected and adapted in human melanoma tumors [4].

In the Introduction, the authors wrote about Rigvir that “most background information regarding the virus is available only in Russian.” While for quite some time most of the background information was available mainly in Latvian and Russian, this has changed in recent years (2015–2022), after the appearance of several publications in English, for example [3,5,7,8,9].

In several instances, the authors speculated about the place of Rigvir^®^ in the phylogenetic chart as a result of the adaptation process. This could be relevant if everything else regarding the different strains were identical, for example, time of collection; however, Figure 1 [1] shows that this is not the case. Therefore, this is pure speculation with no experimental evidence in its support.

## 4. About the Use of Original Rigvir

The authors claim to have used Rigvir, although they provide little information in its support. Rigvir is a prescription medicine that is not provided commercially for non-human use, and the authors did not obtain Rigvir directly from the marketing authorization holder. Therefore, it is not clear whether the authors used genuine Rigvir (see, for example, https://www.rigvir.com/products/beware-of-counterfeits.php (accessed 8 March 2022)) (this might have consequences for the credibility of two publications [1,2]). Several of the details provided may make the reader doubt if the authors did in fact use original Rigvir. For example, the authors (a) did not provide information on the provider; (b) did not provide the batch number(s); (c) did not provide information on their storage conditions, since Rigvir is to be stored at −20 °C; (d) wrote that Rigvir was obtained in an ampule in both [1,2] (ampules are classically sealed using an open flame [10]), while Rigvir is supplied in vials (that are sealed with rubber stoppers and aluminum caps); and (e) wrote that the name of the marketing authorization holder was Sia Latima Ltd., while the real name SIA Latima is printed on both the box and the vial.

## 5. About Missing Information

Furthermore, the source and catalogue numbers of all of the used cells, which are usually provided, are missing: rhabdomyosarcoma (RD), cells, human foreskin fibroblasts (HFF), human bronchial epithelial cell line (16HBE14o), human cervical cancer (HeLa Ohio) cell line, human epithelial lung carcinoma (A549) cell lines, human glioma (U373MG), human hepatocarcinoma (Huh7), human colorectal adenocarcinoma (SW480) and human breast cancer (MCF-7).
